# Supplementary material for: The impact of a disaster medicine clinical training program on medical students’ disaster literacy
Source: PeerJ. 2025 Jan 8;13:e18800. doi: 10.7717/peerj.18800 (PMC11724651; doi:10.7717/peerj.18800)
Supplement: Supplemental Information 5 [file peerj-13-18800-s005.docx]

**Title: English-Language Codebook for Data Files**

**Description:** Below are the codes from the data file translated into English. This codebook has been created to ensure that the Editor, Reviewers, and readers can easily understand the text. The translations correspond directly to the terms used in the raw data files, and each code is aligned with its respective English meaning.

**DATA FİLE NAME_PRE**

1. **ID_Number**
2. **Doğum Yılınız** - **Year of Birth**
3. **Öğrenci No:** - **Student Number**
4. **Cinsiyetiniz** – **Gender**

| Kadın (Female) |
| --- |
| Erkek (Male) |

1. **Afetzede oldunuz mu?** - **Have you been affected by a disaster?**

| Hayır (No) |
| --- |
| Evet (Yes) |

**From this section onward, the scale questions are presented. The answers provided are consistent with the format shown in the table below.**

| 1- Çok zor (“Very Difficult – 1,”) |
| --- |
| 2- Zor (“Difficult – 2,”) |
| 3- Kararsızım (“Undecided – 3,”) |
|  |
| 4- Kolay (“Easy – 4,”) |
| 5- Çok kolay (“Very Easy – 5.”) |

1. 5PRE1_Deprem, sel, heyelan ve kazalar gibi doğa ve insan kaynaklı tehlikelerin nasıl oluştuğu ile ilgili bilgiye ulaşmak - 5PRE1_Accessing information on how natural and human-made hazards such as earthquakes, floods, landslides, and accidents occur
2. 5PRE2_Afetlerin insan, yapı ve çevreye vereceği zararların nasıl azaltılabileceği ile ilgili bilgiye ulaşmak - 5PRE2_Accessing information on how to reduce the damage that disasters can cause to people, structures, and the environment
3. 5PRE3_Sel, fırtına veya bir hortumda zarar görmekten kaçınmak için ihtiyacım olan bilgiye ulaşmak - 5PRE3_Accessing the information I need to avoid harm during a flood, storm, or tornado
4. 5PRE4_Yaşadığım bölge ve coğrafyanın karşılaşabileceği tehlikelerin oluşturabileceği risklerin neler olduğu bilgisine ulaşmak - 5PRE4_Accessing information about the risks posed by hazards in the area and geography I live in
5. 5PRE5_Afet öncesinde, afetlerde ortaya çıkan zararlara karşı yapılabilecek sigortalar hakkında bilgiye ulaşmak - 5PRE5_Accessing information about insurances that can be taken out against the damages caused by disasters
6. 5PRE6_Afet gönüllülerinin eğitimleriyle ilgili bilgilere ulaşmak - 5PRE6_Accessing information on the training of disaster volunteers
7. 5PRE7_Evimi deprem, sel ve yangın gibi tehlikelere karşı yapısal olarak güçlendirmem gerektiğini anlamak - 5PRE7_Understanding the need to structurally strengthen my home against hazards such as earthquakes, floods, and fires
8. 5PRE8_Bir yapının selden zarar görmesini engelleyebilmek için başka bir yere taşındırılma ihtiyacını anlamak - 5PRE8_Understanding the need to relocate a structure to prevent it from being damaged by a flood
9. 5PRE9_Afet öncesinde hazırlanan barınma yerlerinin önemli olduğunu anlamak - 5PRE9_Understanding the importance of shelters prepared before a disaster
10. 5PRE10_Sel gibi afetlerde suya yön vermek için kullanılan kum torbası gibi malzemelerin önemli olduğunu anlamak - 5PRE10_Understanding the importance of materials like sandbags used to direct water during floods
11. 5PRE11_Doğa kaynaklı bazı afetlerde kayıpları azaltmaya yönelik kullanılan erken uyarı sistemlerinin önemini anlamak - 5PRE11_Understanding the importance of early warning systems used to reduce losses in certain natural disasters
12. 5PRE12_Çığ oluşumunu önlemeye karşı kar birikmesini engellemek için patlayıcı kullanma ihtiyacını anlamak - 5PRE12_Understanding the need to use explosives to prevent snow accumulation to avoid avalanches
13. 5PRE13_Afetlerle ilgili hangi eğitime ihtiyacım olduğuna karar vermek - 5PRE13_Deciding which disaster-related training I need
14. 5PRE14_Nerede olursa olsun erken uyarı sistemleri aktif hale geçtiğinde ne yapmam gerektiğine karar vermek - 5PRE14_Deciding what to do when early warning systems are activated, no matter where I am
15. 5PRE15_Yaşadığım ortamda bulunan mobilya ve tablo gibi eşyaların afetler sırasında düşüp devrilerek verebileceği zararı nasıl azaltabileceğime karar vermek - 5PRE15_Deciding how to reduce the damage that items such as furniture and pictures in my living space can cause during a disaster
16. 5PRE16_Afetlere karşı risklerimi/oluşabilecek zararları azaltabilmek için, varsa uygun mali devlet desteğine başvurmak - 5PRE16_Applying for appropriate financial state support if available, to reduce my risks/damages from disasters
17. 5PRE17_Bir afet öncesinde çevremde ki çocuk, yaşlı, engelli veya yardıma ihtiyacı olan vatandaşları tespit etmek - 5PRE17_Identifying children, the elderly, disabled, or others in need of assistance in my surroundings before a disaster
18. 5PRE18_Kurumların halka açık acil durum ve afet planlarına ulaşmak - 5PRE18_Accessing public emergency and disaster plans of institutions
19. 5PRE19_Kurumların halka açık acil durum ve afet tatbikatları hakkındaki bilgilerine ulaşmak - 5PRE19_Accessing public emergency and disaster drills of institutions
20. 5PRE20_Afetlerle ilgili polis, ambulans ve itfaiye gibi ilk müdahale ekiplerinin aldığı eğitimlere ulaşmak - 5PRE20_Accessing the training of first responders like police, ambulance, and fire services related to disasters
21. 5PRE21_Kurumların afetlerle ilgili halka açık genel hazırlık çalışmalarına ulaşmak - 5PRE21_Accessing general preparedness efforts of institutions related to disasters
22. 5PRE22_Afet eğitimlerine neden ihtiyaç olduğunu anlamak - 5PRE22_Understanding why disaster training is necessary
23. 5PRE23_Bir afet anında görevli kişilerin talimatlarına uyum göstermenin önemini anlamak - 5PRE23_Understanding the importance of following the instructions of officials during a disaster
24. 5PRE24_Resmi kurumlar tarafından cep telefonlarına gönderilen afet mesajlarının önemini anlamak - 5PRE24_Understanding the importance of disaster messages sent to mobile phones by official institutions
25. 5PRE25_Benim ve çevremde ki nesnelerin uzun süreli sarsıntısından bir deprem olup olmadığını anlamak - 5PRE25_Understanding whether an earthquake is occurring based on the prolonged shaking of myself and objects around me
26. 5PRE26_Afet öncesinde resmi kurumlar tarafından yapılan uyarı ve ikazların önemini anlamak - 5PRE26_Understanding the importance of warnings and alerts made by official institutions before a disaster
27. 5PRE27_Afetlere hazırlık amacıyla yapılan tatbikatlarda aile bireylerinin rol ve sorumluluklarının nasıl belirleneceğine karar vermek - 5PRE27_Deciding how to allocate roles and responsibilities among family members during disaster preparedness drills
28. 5PRE28_Afet çantasında neler olması gerektiğine karar vermek - 5PRE28_Deciding what should be included in a disaster kit
29. 5PRE29_Afete neden olan tehlikelerle ilgili medyada yer alan bilgilerin güvenirliğini değerlendirmek - 5PRE29_Evaluating the reliability of information in the media regarding hazards causing disasters
30. 5PRE30_Aile bireyleriyle aile afet planı hazırlamak - 5PRE30_Preparing a family disaster plan with family members
31. 5PRE31_Aile afet planı içerisinde hazırlanan tatbikat planlarını aile bireyleriyle uygulamak - 5PRE31_Implementing drill plans prepared within the family disaster plan with family members
32. 5PRE32_İlk yardım, yangın söndürme ve temel arama kurtarma eğitimlerine başvurmak - 5PRE32_Applying for first aid, firefighting, and basic search and rescue training
33. 5PRE33_Kurumlar tarafından tehlikeyle ilgili uyarı ve ikazlar afet vurmadan önce hemen yapılabilirse, alandan tahliye bilgisine ulaşmak - 5PRE33_Accessing information on evacuating the area if warnings and alerts are made immediately before a disaster by institutions
34. 5PRE34_Yerel kurumlar tarafından afet anında kullanılmak üzere park, bahçe gibi kamusal alanlara yerleştirilen afet ekipmanları bilgisine ulaşmak - 5PRE34_Accessing information about disaster equipment placed in public areas like parks and gardens for use during disasters
35. 5PRE35_Toplumda sel veya fırtına gibi bir tehlikenin erken uyarısı yapıldığında afetten korunmak için gerekli bilgiye ulaşmak - 5PRE35_Accessing the information needed to protect yourself from a disaster when early warnings of hazards like floods or storms are made in the community
36. 5PRE36_Bulunduğum ortamı terk edebileceğim bir kaçış planının ne kadar önemli olduğunu anlamak - 5PRE36_Understanding the importance of having an escape plan from my location
37. **5PRE37_Kurumların ilaç ve tıbbi malzeme, gıda ve giysi gibi öğeler için oluşturdukları afet ekipman kitlerinin önemini
38. 5PRE37_Kurumların ilaç ve tıbbi malzeme, gıda ve giysi gibi öğeler için oluşturdukları afet ekipman kitlerinin önemini anlamak - 5PRE37_Understanding the importance of disaster equipment kits prepared by institutions for items like medicine, food, and clothing
39. 5PRE38_Bir sel afeti öncesinde çevrenin sular altında kalmasını kum torbaları ile engelleyebilmenin önemli olduğunu anlamak - 5PRE38_Understanding the importance of preventing the environment from being submerged by water with sandbags before a flood
40. 5PRE39_Arama kurtarma çalışmalarında ilk 72 saatin önemli olduğunu anlamak - 5PRE39_Understanding the importance of the first 72 hours in search and rescue operations
41. 5PRE40_Ambulans, itfaiye, emniyet veya diğer yardım ekiplerinin boş yere meşgul edilmemesi gerektiğini anlamak - 5PRE40_Understanding that emergency response teams like ambulances, fire brigades, and police should not be occupied unnecessarily
42. 5PRE41_Bir afet anında ihtiyaç halinde hangi kurumla temasa geçebileceğime karar vermek - 5PRE41_Deciding which institution to contact in case of need during a disaster
43. 5PRE42_Afet anında ikincil tehlikelerden korunmak için yapmam gerekenleri (bölgeyi terk etme veya bir aşı yaptırmak gibi) değerlendirmek - 5PRE42_Evaluating what I need to do to protect myself from secondary hazards during a disaster (e.g., evacuating the area or getting a vaccine)
44. 5PRE43_Yaralanan bir kişinin ilk yardıma ihtiyacı olup olmadığına karar vermek - 5PRE43_Deciding whether an injured person needs first aid
45. 5PRE44_Afet anında en yakın afet toplanma alanına gitmek - 5PRE44_Going to the nearest disaster assembly area during a disaster
46. 5PRE45_Tahliye edilme talimatlarına uyum göstermek - 5PRE45_Complying with evacuation instructions
47. 5PRE46_Bir afet sonrası iyileştirme çalışmaları kapsamında devreye giren afet planlarına ulaşmak - 5PRE46_Accessing disaster plans implemented during post-disaster recovery efforts
48. 5PRE47_Bir afet sonrasında yapı yönetmelikleriyle ilgili düzenlenen güncel bilgilere ulaşmak - 5PRE47_Accessing updated information on building regulations after a disaster
49. 5PRE48_Bir afet yerinde oluşturulan afet ve acil durum yönetim merkezi kadrosunun hangi birimlerden oluştuğu bilgisine ulaşmak - 5PRE48_Accessing information on which units the disaster and emergency management center consists of at a disaster site
50. 5PRE49_Bir afet sonrasında ulaşım gibi kamusal hizmetlerin faal olup olmadığı bilgisine ulaşmak - 5PRE49_Accessing information on whether public services like transportation are operational after a disaster
51. 5PRE50_Bir afet sonrasında oluşan fiziksel ve ruhsal sağlık sorunlarıyla nasıl mücadele edileceği ile ilgili bilgiye ulaşmak - 5PRE50_Accessing information on how to cope with physical and mental health issues that arise after a disaster
52. 5PRE51_Bir afet sonrasında yaşadığım yapının hasar değerlendirmesini yaptırmanın önemini anlamak - 5PRE51_Understanding the importance of having my home assessed for damage after a disaster
53. 5PRE52_Resmi ve özel kurum ile toplumu temsil eden kişilerin afetler sonrasında işbirliği içinde çalışmalarının önemini anlamak - 5PRE52_Understanding the importance of cooperation between public and private institutions and community representatives after a disaster
54. 5PRE53_Bir afet sonrasında çocuk, yaşlı veya engelliler gibi özel ihtiyaç sahibi vatandaşların yardım gereksinimlerinin neler olabileceğini anlamak - 5PRE53_Understanding the needs of special needs citizens like children, the elderly, or the disabled after a disaster
55. 5PRE54_Bir afet sonrasında kültürel eserleri korumanın toplum açısından önemini anlamak - 5PRE54_Understanding the importance of protecting cultural assets after a disaster
56. 5PRE55_Bir afet sonrasında iyileşmeyi hızlandırmak için yetkililerle işbirliği içinde olmanın önemini değerlendirmek - 5PRE55_Evaluating the importance of cooperating with authorities to accelerate recovery after a disaster
57. 5PRE56_Bir afet sonrasında gıda, barınma ve sağlık gibi temel ihtiyaçlarımı mevcut şartlara en uygun şekilde değerlendirmek - 5PRE56_Evaluating my basic needs like food, shelter, and health according to existing conditions after a disaster
58. 5PRE57_Afetlerde zarar gören doğal kaynakların bildirimini hangi kurumlara yapabileceğimi değerlendirmek - 5PRE57_Evaluating which institutions I can report damage to natural resources caused by disasters
59. 5PRE58_Bir afet sonrasında kullanılmak üzere devletin ayırdığı mali kaynaklardan yararlanmak için ilgili birimlere başvurmak - 5PRE58_Applying to the relevant units to benefit from the financial resources allocated by the state for use after disasters
60. 5PRE59_Afetlerde hasar gören bir yapının onarımını yönetmeliklere uygun bir şekilde yaptırmak - 5PRE59_Ensuring that the repair of a structure damaged in disasters is done according to regulations
61. 5PRE60_Afetler sırasında ulaşım, enerji ve kanalizasyon gibi alt ve üst yapı sistemlerinde hasar meydana gelirse, yerel kurumların ilgili birimlerini bilgilendirmek - 5PRE60_Informing the relevant units of local institutions if there is damage to infrastructure systems like transportation, energy, and sewage during disasters

**DATA FİLE NAME_POST**

ID - ID

1. Doğum Yılınız - Year of Birth
2. Öğrenci No: - Student Number
3. Cinsiyetiniz - Gender
4. Afetzede oldunuz mu? - Have you been affected by a disaster?
5. 5POST1_DePREM, sel, heyelan ve kazalar gibi doğa ve insan kaynaklı tehlikelerin nasıl oluştuğu ile ilgili bilgiye ulaşmak - 5POST1_Accessing information on how natural and human-made hazards such as earthquakes, floods, landslides, and accidents occur
6. 5POST2_Afetlerin insan, yapı ve çevreye vereceği zararların nasıl azaltılabileceği ile ilgili bilgiye ulaşmak - 5POST2_Accessing information on how to reduce the damage that disasters can cause to people, structures, and the environment
7. 5POST3_Sel, fırtına veya bir hortumda zarar görmekten kaçınmak için ihtiyacım olan bilgiye ulaşmak - 5POST3_Accessing the information I need to avoid harm during a flood, storm, or tornado
8. 5POST4_Yaşadığım bölge ve coğrafyanın karşılaşabileceği tehlikelerin oluşturabileceği risklerin neler olduğu bilgisine ulaşmak - 5POST4_Accessing information about the risks posed by hazards in the area and geography I live in
9. 5POST5_Afet öncesinde, afetlerde ortaya çıkan zararlara karşı yapılabilecek sigortalar hakkında bilgiye ulaşmak - 5POST5_Accessing information about insurances that can be taken out against the damages caused by disasters
10. 5POST6_Afet gönüllülerinin eğitimleriyle ilgili bilgilere ulaşmak - 5POST6_Accessing information on the training of disaster volunteers
11. 5POST7_Evimi deprem, sel ve yangın gibi tehlikelere karşı yapısal olarak güçlendirmem gerektiğini anlamak - 5POST7_Understanding the need to structurally strengthen my home against hazards such as earthquakes, floods, and fires
12. 5POST8_Bir yapının selden zarar görmesini engelleyebilmek için başka bir yere taşındırılma ihtiyacını anlamak - 5POST8_Understanding the need to relocate a structure to prevent it from being damaged by a flood
13. 5POST9_Afet öncesinde hazırlanan barınma yerlerinin önemli olduğunu anlamak - 5POST9_Understanding the importance of shelters prepared before a disaster
14. 5POST10_Sel gibi afetlerde suya yön vermek için kullanılan kum torbası gibi malzemelerin önemli olduğunu anlamak - 5POST10_Understanding the importance of materials like sandbags used to direct water during floods
15. 5POST11_Doğa kaynaklı bazı afetlerde kayıpları azaltmaya yönelik kullanılan erken uyarı sistemlerinin önemini anlamak - 5POST11_Understanding the importance of early warning systems used to reduce losses in certain natural disasters
16. 5POST12_Çığ oluşumunu önlemeye karşı kar birikmesini engellemek için patlayıcı kullanma ihtiyacını anlamak - 5POST12_Understanding the need to use explosives to prevent snow accumulation to avoid avalanches
17. 5POST13_Afetlerle ilgili hangi eğitime ihtiyacım olduğuna karar vermek - 5POST13_Deciding which disaster-related training I need
18. 5POST14_Nerede olursa olsun erken uyarı sistemleri aktif hale geçtiğinde ne yapmam gerektiğine karar vermek - 5POST14_Deciding what to do when early warning systems are activated, no matter where I am
19. 5POST15_Yaşadığım ortamda bulunan mobilya ve tablo gibi eşyaların afetler sırasında düşüp devrilerek verebileceği zararı nasıl azaltabileceğime karar vermek - 5POST15_Deciding how to reduce the damage that items such as furniture and pictures in my living space can cause during a disaster
20. 5POST16_Afetlere karşı risklerimi/oluşabilecek zararları azaltabilmek için, varsa uygun mali devlet desteğine başvurmak - 5POST16_Applying for appropriate financial state support if available, to reduce my risks/damages from disasters
21. 5POST17_Bir afet öncesinde çevremdeki çocuk, yaşlı, engelli veya yardıma ihtiyacı olan vatandaşları tespit etmek - 5POST17_Identifying children, the elderly, disabled, or others in need of assistance in my surroundings before a disaster
22. 5POST18_Kurumların halka açık acil durum ve afet planlarına ulaşmak - 5POST18_Accessing public emergency and disaster plans of institutions
23. 5POST19_Kurumların halka açık acil durum ve afet tatbikatları hakkındaki bilgilerine ulaşmak - 5POST19_Accessing public emergency and disaster drills of institutions
24. 5POST20_Afetlerle ilgili polis, ambulans ve itfaiye gibi ilk müdahale ekiplerinin aldığı eğitimlere ulaşmak - 5POST20_Accessing the training of first responders like police, ambulance, and fire services related to disasters
25. 5POST21_Kurumların afetlerle ilgili halka açık genel hazırlık çalışmalarına ulaşmak - 5POST21_Accessing general preparedness efforts of institutions related to disasters
26. 5POST22_Afet eğitimlerine neden ihtiyaç olduğunu anlamak - 5POST22_Understanding why disaster training is necessary
27. 5POST23_Bir afet anında görevli kişilerin talimatlarına uyum göstermenin önemini anlamak - 5POST23_Understanding the importance of following the instructions of officials during a disaster
28. 5POST24_Resmi kurumlar tarafından cep telefonlarına gönderilen afet mesajlarının önemini anlamak - 5POST24_Understanding the importance of disaster messages sent to mobile phones by official institutions
29. 5POST25_Benim ve çevremdeki nesnelerin uzun süreli sarsıntısından bir deprem olup olmadığını anlamak - 5POST25_Understanding whether an earthquake is occurring based on the prolonged shaking of myself and objects around me
30. 5POST26_Afet öncesinde resmi kurumlar tarafından yapılan uyarı ve ikazların önemini anlamak - 5POST26_Understanding the importance of warnings and alerts made by official institutions before a disaster
31. 5POST27_Afetlere hazırlık amacıyla yapılan tatbikatlarda aile bireylerinin rol ve sorumluluklarının nasıl belirleneceğine karar vermek - 5POST27_Deciding how to allocate roles and responsibilities among family members during disaster preparedness drills
32. 5POST28_Afet çantasında neler olması gerektiğine karar vermek - 5POST28_Deciding what should be included in a disaster kit
33. 5POST29_Afete neden olan tehlikelerle ilgili medyada yer alan bilgilerin güvenirliğini değerlendirmek - 5POST29_Evaluating the reliability of information in the media regarding hazards causing disasters
34. 5POST30_Aile bireyleriyle aile afet planı hazırlamak - 5POST30_Preparing a family disaster plan with family members
35. 5POST31_Aile afet planı içerisinde hazırlanan tatbikat planlarını aile bireyleriyle uygulamak - 5POST31_Implementing drill plans prepared within the family disaster plan with family members
36. 5POST32_İlk yardım, yangın söndürme ve temel arama kurtarma eğitimlerine başvurmak - 5POST32_Applying for first aid, firefighting, and basic search and rescue training
37. 5POST33_Kurumlar tarafından tehlikeyle ilgili uyarı ve ikazlar afet vurmadan önce hemen yapılabilirse, alandan tahliye bilgisine ulaşmak - 5POST33_Accessing information on evacuating the area if warnings and alerts are made immediately before a disaster by institutions
38. 5POST34_Yerel kurumlar tarafından afet anında kullanılmak üzere park, bahçe gibi kamusal alanlara yerleştirilen afet ekipmanları bilgisine ulaşmak - 5POST34_Accessing information about disaster equipment placed in public areas like parks and gardens for use during disasters
39. 5POST35_Toplumda sel veya fırtına gibi bir tehlikenin erken uyarısı yapıldığında afetten korunmak için gerekli bilgiye ulaşmak - 5POST35_Accessing the information needed to protect yourself from a disaster when early warnings of hazards like floods or storms are made in the community
40. 5POST36_Bulunduğum ortamı terk edebileceğim bir kaçış planının ne kadar önemli olduğunu anlamak - 5POST36_Understanding the importance of having an escape plan from my location
41. 5POST37_Kurumların ilaç ve tıbbi malzeme, gıda ve giysi gibi öğeler için oluşturdukları afet ekipman kitlerinin önemini anlamak - 5POST37_Understanding the importance of disaster equipment kits prepared by institutions for items like medicine, food, and clothing
42. 5POST38_Bir sel afeti öncesinde çevrenin sular altında kalmasını kum torbaları ile engelleyebilmenin önemli olduğunu anlamak - 5POST38_Understanding the importance of preventing the environment from being submerged by water with sandbags before a flood
43. 5POST39_Arama kurtarma çalışmalarında ilk 72 saatin önemli olduğunu anlamak - 5POST39_Understanding the importance of the first 72 hours in search and rescue operations
44. 5POST40_Ambulans, itfaiye, emniyet veya diğer yardım ekiplerinin boş yere meşgul edilmemesi gerektiğini anlamak - 5POST40_Understanding that emergency response teams like ambulances, fire brigades, and police should not be occupied unnecessarily
45. 5POST41_Bir afet anında ihtiyaç halinde hangi kurumla temasa geçebileceğime karar vermek - 5POST41_Deciding which institution to contact in case of need during a disaster
46. 5POST42_Afet anında ikincil tehlikelerden korunmak için yapmam gerekenleri (bölgeyi terk etme veya bir aşı yaptırmak gibi) değerlendirmek - 5POST42_Evaluating what I need to do to protect myself from secondary hazards during a disaster (e.g., evacuating the area or getting a vaccine)
47. 5POST43_Yaralanan bir kişinin ilk yardıma ihtiyacı olup olmadığına karar vermek - 5POST43_Deciding whether an injured person needs first aid
48. 5POST44_Afet anında en yakın afet toplanma alanına gitmek - 5POST44_Going to the nearest disaster assembly area during a disaster
49. 5POST45_Tahliye edilme talimatlarına uyum göstermek - 5POST45_Complying with evacuation instructions
50. 5POST46_Bir afet sonrası iyileştirme çalışmaları kapsamında devreye giren afet planlarına ulaşmak - 5POST46_Accessing disaster plans implemented during post-disaster recovery efforts
51. 5POST47_Bir afet sonrasında yapı yönetmelikleriyle ilgili düzenlenen güncel bilgilere ulaşmak - 5POST47_Accessing updated information on building regulations after a disaster
52. 5POST48_Bir afet yerinde oluşturulan afet ve acil durum yönetim merkezi kadrosunun hangi birimlerden oluştuğu bilgisine ulaşmak - 5POST48_Accessing information on which units the disaster and emergency management center consists of at a disaster site
53. 5POST49_Bir afet sonrasında ulaşım gibi kamusal hizmetlerin faal olup olmadığı bilgisine ulaşmak - 5POST49_Accessing information on whether public services like transportation are operational after a disaster
54. 5POST50_Bir afet sonrasında oluşan fiziksel ve ruhsal sağlık sorunlarıyla nasıl mücadele edileceği ile ilgili bilgiye ulaşmak - 5POST50_Accessing information on how to cope with physical and mental health issues that arise after a disaster
55. 5POST51_Bir afet sonrasında yaşadığım yapının hasar değerlendirmesini yaptırmanın önemini anlamak - 5POST51_Understanding the importance of having my home assessed for damage after a disaster
56. 5POST52_Resmi ve özel kurum ile toplumu temsil eden kişilerin afetler sonrasında işbirliği içinde çalışmalarının önemini anlamak - 5POST52_Understanding the importance of cooperation between public and private institutions and community representatives after a disaster
57. 5POST53_Bir afet sonrasında çocuk, yaşlı veya engelliler gibi özel ihtiyaç sahibi vatandaşların yardım gereksinimlerinin neler olabileceğini anlamak - 5POST53_Understanding the needs of special needs citizens like children, the elderly, or the disabled after a disaster
58. 5POST54_Bir afet sonrasında kültürel eserleri korumanın toplum açısından önemini anlamak - 5POST54_Understanding the importance of protecting cultural assets after a disaster
59. 5POST55_Bir afet sonrasında iyileşmeyi hızlandırmak için yetkililerle işbirliği içinde olmanın önemini değerlendirmek - 5POST55_Evaluating the importance of cooperating with authorities to accelerate recovery after a disaster
60. 5POST56_Bir afet sonrasında gıda, barınma ve sağlık gibi temel ihtiyaçlarımı mevcut şartlara en uygun şekilde değerlendirmek - 5POST56_Evaluating my basic needs like food, shelter, and health according to existing conditions after a disaster
61. 5POST57_Afetlerde zarar gören doğal kaynakların bildirimini hangi kurumlara yapabileceğimi değerlendirmek - 5POST57_Evaluating which institutions I can report damage to natural resources caused by disasters
62. 5POST58_Bir afet sonrasında kullanılmak üzere devletin ayırdığı mali kaynaklardan yararlanmak için ilgili birimlere başvurmak - 5POST58_Applying to the relevant units to benefit from the financial resources allocated by the state for use after disasters
63. 5POST59_Afetlerde hasar gören bir yapının onarımını yönetmeliklere uygun bir şekilde yaptırmak - 5POST59_Ensuring that the repair of a structure damaged in disasters is done according to regulations
64. 5POST60_Afetler sırasında ulaşım, enerji ve kanalizasyon gibi alt ve üst yapı sistemlerinde hasar meydana gelirse, yerel kurumların ilgili birimlerini bilgilendirmek - 5POST60_Informing the relevant units of local institutions if there is damage to infrastructure systems like transportation, energy, and sewage during disasters
