# Supplementary material for: The impact of a disaster medicine clinical training program on medical students’ disaster literacy
Source: PeerJ. 2025 Jan 8;13:e18800. doi: 10.7717/peerj.18800 (PMC11724651; doi:10.7717/peerj.18800)
Supplement: Supplemental Information 6 [file peerj-13-18800-s006.docx]

*DLS Scale*

Below are some terms and phrases regarding disaster literacy. Read the terms "disaster, hazard and risk" before answering the questions. Then, in light of these terms, please continue to answer the expressions in the table. You can give your most appropriate answers by putting crosses (x) on expressions as "Very difficult – 1", "Difficult – 2", "Undecided – 3", "Easy – 4" and "Very easy – 5". Reading all of the expressions carefully and answering correctly are important for the value of the study.

Definitions:

***Disaster:*** *A natural, technological or human-induced event that creates physical, economic and social losses for all or certain segments of the society and halts or disrupts normal life and human activities, as a consequence of which the affected society does not have enough capacity to cope.*

***Hazard:*** *Physical events and facts caused by nature, technology or human beings, which are life-threatening, appear in a certain time or geography, and have the potential to harm the socioeconomic order and activities of the society along with natural environment and natural, historical and cultural resources.*

***Risk:*** *The possibility of loss of values such as life, property, and economic and environmental values that an event can cause under certain conditions and environments.*

| **No** | **1 – Very difficult**  **2 – Difficult**  **3 – Undecided**  **4 – Easy**  **5 – Very Easy** | |
| --- | --- | --- |
| **1** | To reach the information about how natural and human-induced hazards, such as earthquakes, floods, landslides and accidents, occur | ➀ ➁ ➂ ➃ ➄ |
| **2** | To learn how to reduce the harm of disasters to people, structures and the environment | ➀ ➁ ➂ ➃ ➄ |
| **3** | To reach the information needed to avoid damage caused by flooding, storms or tornadoes | ➀ ➁ ➂ ➃ ➄ |
| **4** | To reach the knowledge of what risks can cause hazard in the region and geography I live | ➀ ➁ ➂ ➃ ➄ |
| **5** | To reach the information about insurances that can be done against potential damages caused by disasters before they occur | ➀ ➁ ➂ ➃ ➄ |
| **6** | To reach the information about the trainings of disaster volunteers | ➀ ➁ ➂ ➃ ➄ |
| **7** | To understand that I need to structurally strengthen my house against hazards such as earthquake, flood and fire | ➀ ➁ ➂ ➃ ➄ |
| **8** | To understand the necessity to move to another place in order to prevent flood damage | ➀ ➁ ➂ ➃ ➄ |
| **9** | To understand the importance of sheltering areas before the disaster | ➀ ➁ ➂ ➃ ➄ |
| **10** | To understand the importance of materials such as sandbags used to lead water in disasters such as flood | ➀ ➁ ➂ ➃ ➄ |
| **11** | To understand the importance of early warning systems used to reduce losses in some natural disasters | ➀ ➁ ➂ ➃ ➄ |
| **12** | To understand the need to use explosives to prevent snow accumulation and a potential avalanche formation | ➀ ➁ ➂ ➃ ➄ |
| **13** | To decide what training I need on disasters | ➀ ➁ ➂ ➃ ➄ |
| **No** | **1 – Very Difficult**  **2 – Difficult**  **3 – Undecided**  **4 – Easy**  **5 – Very Easy** | |
| **14** | To decide what to do when early warning systems become active wherever I am | ➀ ➁ ➂ ➃ ➄ |
| **15** | To decide how to reduce the damage that items such as furniture and paintings in my environment can cause by falling down during disasters | ➀ ➁ ➂ ➃ ➄ |
| **16** | If there is any, applying for an appropriate financial government support to reduce the risks/damages that may occur during disasters. | ➀ ➁ ➂ ➃ ➄ |
| **17** | To identify children, the elderly, the disabled or people in need of help before a disaster | ➀ ➁ ➂ ➃ ➄ |
| **18** | To reach the public emergency and disaster plans of institutions | ➀ ➁ ➂ ➃ ➄ |
| **19** | To reach the information about public emergency and disaster drills | ➀ ➁ ➂ ➃ ➄ |
| **20** | To access the disaster trainings received by emergency responders such as police, ambulance and fire fighters | ➀ ➁ ➂ ➃ ➄ |
| **21** | To reach the public preparatory works of institutions on disasters | ➀ ➁ ➂ ➃ ➄ |
| **22** | To understand why disaster training is necessary | ➀ ➁ ➂ ➃ ➄ |
| **23** | To understand the importance of complying with the instructions of people in charge during a disaster | ➀ ➁ ➂ ➃ ➄ |
| **24** | To understand the importance of disaster messages sent by government agencies to mobile phones | ➀ ➁ ➂ ➃ ➄ |
| **25** | To understand whether there is an earthquake from objects around me and my long-term shock | ➀ ➁ ➂ ➃ ➄ |
| **26** | To understand the importance of warnings made by government agencies before disasters | ➀ ➁ ➂ ➃ ➄ |
| **27** | To decide how to determine the role and responsibilities of family members in drills for disaster preparedness | ➀ ➁ ➂ ➃ ➄ |
| **28** | To decide which training courses I need on disasters | ➀ ➁ ➂ ➃ ➄ |
| **29** | To decide what should be in an emergency backpack | ➀ ➁ ➂ ➃ ➄ |
| **30** | To assess the reliability of media information about hazards that cause a disaster | ➀ ➁ ➂ ➃ ➄ |
| **31** | To prepare a disaster plan with family members | ➀ ➁ ➂ ➃ ➄ |
| **32** | To implement drill plans prepared within the family disaster plan with family members | ➀ ➁ ➂ ➃ ➄ |
| **33** | To apply to first aid, firefighting and basic search and rescue trainings | ➀ ➁ ➂ ➃ ➄ |
| **34** | If warnings about hazard can be made immediately by the agencies before the disaster occurs, to reach the evacuation information from the area | ➀ ➁ ➂ ➃ ➄ |
| **35** | To access the knowledge of disaster equipment placed in public areas by local agencies, such as parks and gardens, for use in a disaster | ➀ ➁ ➂ ➃ ➄ |
| **36** | To reach the necessary information for protection from disasters when an early warning of a hazard such as flood or storm is made publicly | ➀ ➁ ➂ ➃ ➄ |
| **No** | **1 – Very Difficult**  **2 – Difficult**  **3 – Undecided**  **4 – Easy**  **5 – Very Easy** | |
| **37** | To understand how important an escape plan is to leave the environment I am in | ➀ ➁ ➂ ➃ ➄ |
| **38** | To understand the importance of disaster equipment kits that institutions create for items such as medicines, medical supplies, food and clothing | ➀ ➁ ➂ ➃ ➄ |
| **39** | To understand that it is important to be able to prevent the environment from flooding with sandbags before a flood disaster | ➀ ➁ ➂ ➃ ➄ |
| **40** | To understand that the first 72 hours are important in search and rescue efforts | ➀ ➁ ➂ ➃ ➄ |
| **41** | To understand that ambulances, fire fighters, police or other aid teams should not be kept busy in vain | ➀ ➁ ➂ ➃ ➄ |
| **42** | To decide which institution I can contact if needed in the event of a disaster | ➀ ➁ ➂ ➃ ➄ |
| **43** | To assess what I need to do (such as leaving the area or getting vaccinated) to avoid secondary hazards in a disaster | ➀ ➁ ➂ ➃ ➄ |
| **44** | To decide whether an injured person needs first aid or not | ➀ ➁ ➂ ➃ ➄ |
| **45** | To go to the nearest emergency gathering area at the time of a disaster | ➀ ➁ ➂ ➃ ➄ |
| **46** | To comply with evacuation instructions | ➀ ➁ ➂ ➃ ➄ |
| **47** | To reach disaster plans that come into effect as part of a post-disaster recovery efforts | ➀ ➁ ➂ ➃ ➄ |
| **48** | To get up-to-date information about building regulations after a disaster | ➀ ➁ ➂ ➃ ➄ |
| **49** | To reach the information about which units are included in the disaster and emergency management center staff created at a disaster site | ➀ ➁ ➂ ➃ ➄ |
| **50** | To get the information about whether public services such as transportation are active after a disaster | ➀ ➁ ➂ ➃ ➄ |
| **51** | To reach the information on how to face with physical and mental health problems after a disaster | ➀ ➁ ➂ ➃ ➄ |
| **52** | To understand the importance of making the damage assessment of the building I live in after a disaster | ➀ ➁ ➂ ➃ ➄ |
| **53** | To understand the importance of working in cooperation with the official and private institutions and people representing the community in the wake of disasters | ➀ ➁ ➂ ➃ ➄ |
| **54** | To understand the needs of especially needy citizens, such as children, the elderly or the disabled, after a disaster | ➀ ➁ ➂ ➃ ➄ |
| **55** | To understand the importance of protecting cultural artifacts for the society after a disaster | ➀ ➁ ➂ ➃ ➄ |
| **56** | To assess the importance of cooperating with authorities to speed up recovery in the wake of a disaster | ➀ ➁ ➂ ➃ ➄ |
| **57** | To evaluate my basic needs such as food, housing and health in the best way possible to meet the current conditions after a disaster | ➀ ➁ ➂ ➃ ➄ |
| **58** | To assess to which institutions natural resources damaged in disasters can be reported | ➀ ➁ ➂ ➃ ➄ |
| **59** | To contact the relevant units to take advantage of the government's financial resources for use after a disaster | ➀ ➁ ➂ ➃ ➄ |
| **No** | **1 – Very Difficult**  **2 – Difficult**  **3 – Undecided**  **4 – Easy**  **5 – Very Easy** | |
| **60** | To repair a damaged structure in disasters in accordance with regulations | ➀ ➁ ➂ ➃ ➄ |
| **61** | To inform the relevant units of local institutions if damage occurs in infrastructure and superstructure systems such as transportation, energy and sewerage during disasters | ➀ ➁ ➂ ➃ ➄ |

*Thank you for your participation.*
